# The effect of acupuncture on condition being studied emotional disorders in patients with postpartum: A protocol for systematic review and meta-analysis

**DOI:** 10.1097/MD.0000000000028669

**Published:** 2022-01-28

**Authors:** Ning Luo, Yiyi Wang, Yunfan Xia, Mingqi Tu, Xiaoting Wu, Xiaomei Shao, Jianqiao Fang

**Affiliations:** The Third Clinical Medical College of Zhejiang Chinese Medical University, Hangzhou City, Zhejiang Province, China.

**Keywords:** acupuncture, emotional disorders, meta-analysis, perinatal women, postpartum

## Abstract

**Background:**

As one of the common postpartum diseases, postpartum emotional disorders (PEDs) mainly include postpartum depression, postpartum anxiety, posttraumatic stress disorder, and obsessive-compulsive disorder, which significantly affect the patient's quality of life. Acupuncture has been widely used as a popular alternative complementary therapy for the treatment of PEDs. Nevertheless, its effectiveness and safety remain uncertain. Hence, the first systematic review and meta-analysis will be urgently executed to explore the effectiveness and safety of acupuncture in the treatment of PEDs.

**Methods:**

Eight databases will be searched, including the PubMed, Web of Science, EMBASE, the Cochrane Central Register of Controlled Trials, Chinese National Knowledge Infrastructure, Chinese Biomedical Literature Database, Wanfang Database, and Technology Periodical Database. Only randomized controlled trials of acupuncture for PEDs will be considered. The languages are limited to English and Chinese. All publications were retrieved by 2 researchers independently. Assessment of the Edinburgh Postpartum Depression Scale will be dedicated as a primary outcome, and secondary outcomes include the Hamilton Anxiety Inventory, the Hamilton Depression Inventory, the Orientation to Life Questionnaire (sense of coherence 29-item scale), and adverse effects of acupuncture. The Cochrane Risk of Bias tool will be used to assess the quality of the eligible publications. Additionally, the level of evidence for results will be evaluated by using the Grades of Recommendation, Assessment, Development, and Evaluation method. All data will be analyzed statistically by using RevMan V.5.3 software.

**Results:**

This study will provide a high level of the evidence-based basis for the effectiveness and safety of acupuncture in the treatment of PEDs.

**Conclusion:**

The findings of this study will assess the safety, efficacy, and adverse effects of acupuncture in the treatment of PEDs.

**Ethics and dissemination:**

No ethical approval is required as patient data will not be collected. In addition, the results of this meta-analysis will be disseminated through publication in peer-reviewed scholarly journals or relevant academic conferences.

**Registration number:**

INPLASY 2021120091.

## 1 Introduction

Postpartum emotional disorders (PEDs) are psychological disorders that occur specifically during the period of postpartum, including postpartum depression (PPD), postpartum anxiety, postpartum blues, posttraumatic stress disorder, obsessive-compulsive disorder.^[1,2]^ Among them, PPD is a typical manifestation of PEDs and has been concerned by domestic and international research, with a prevalence of about 40%.^[3]^ Moreover, and it is widely suggested believed that postpartum anxiety and depression exist the have co-morbid characteristics.^[4,5]^ The cause of PEDs may be the significant effects of postnatal hormonal fluctuations on self-regulation, empathy, and emotion-related neural activity.^[6]^ Worryingly, approximately 27% of the postpartum women suffer from PEDs,^[7]^ nevertheless, nearly 50% of patients do not seek help from professionals, family, or friends, it often had been overlooked.^[8]^ Hence, the both physical and mental health of the maternal and infant can be affected. Besides, the relationships of parent-child and family were influenced significantly as well.^[9]^ Severely, suicide as a result of PEDs is one of the leading causes of maternal death.^[10,11]^

The most commonly prescribed drugs for PEDs remain to be antidepressants, anxiolytics, psychostimulants, and antipsychotics.^[2,12]^ However, drug-induced side effects, such as dizziness, inattention, and ataxia, often lead to poor patient compliance, which reduces the effectiveness of treatment.^[13–15]^ In particular, some drugs expose infants to the risk of disease through breastfeeding.^[16,17]^ Additionally, Psychological interventions are also one of the treatments for PEDs but are limited by the lack of providers and financial resources.^[18]^ Hence, an alternative therapy is urgently warranted to treat PEDs.

Robust evidence demonstrated that the effectiveness of acupuncture in treating emotional disorders such as depression and anxiety.^[19–22]^ The mechanism of acupuncture for emotional disorders may be regulation of the HPA axis and enhancement of 5 -/5-hydroxytryptamine 1A receptor levels in the hippocampus to modulate the expression of related emotional genes.^[23–25]^ Decreased pro-inflammatory cytokines in hippocampus, prefrontal cortex, and serum may be another mechanism for acupuncture in the treatment of PEDs.^[26]^ However, the effectiveness of acupuncture for PEDs remains largely uncertain and the relevant evidence is deficient. Accordingly, the first systematic review and meta-analysis will be conducted to investigate the efficacy and safety of acupuncture in the treatment of PEDs.

## 2 Objectives

This study will provide the first systematic review and meta-analysis of comprehensive data from the eight databases to explore the effectiveness and safety of acupuncture in the treatment of PEDs.

## 3 Methods

This program has been registered in International Platform of Registered Systematic Review and Meta-analysis Protocols (INPLASY 2021120091) and completed by the Preferred Reporting Items for Systems Evaluation and Meta-Analysis Program.^[27]^ Any revisions to the program will be documented on the INPLASY platform (https://inplasy.com/).

### 3.1 Inclusion criteria for literature selection

#### 3.1.1 Types of studies

Only randomized controlled trials (RCTs) in the treatment acupuncture for PEDs will be included, with language restrictions to Chinese and English. Besides, eligible publications that randomization was mentioned will also be considered. Whereas studies that used randomization methods incorrectly and other non-RCTs will be excluded.

#### 3.1.2 Types of participants

Participants must be diagnosed with PEDs. No restrictions on age, nationality, and race. Diagnosis of the PEDs is primarily confirmed by a comprehensive clinical interview and assessment of classification systems such as the Diagnostic and Statistical Manual of Mental Disorders^[28]^ or The International Statistical Classification of Diseases and Related Health Problems, 11th revision.^[29]^

#### 3.1.3 Types of intervention

The intervention methods, including manual acupuncture, electroacupuncture, auricular acupuncture, scalp acupuncture, and warm needle acupuncture will be considered. In addition, acupuncture as the sole treatment or combined with other therapies will be also included in this study.

#### 3.1.4 Type of comparators or control

Comparison interventions will be sham acupuncture, noninvasive placebo acupuncture, no treatment, waiting list membership, conventional medication, psychotherapy, physical therapy, and other active treatment methods. In addition, studies comparing the efficacy of different forms of acupuncture and different acupuncture points will be excluded. Combination treatment modalities that cannot assess the efficacy of acupuncture will also not be included.

### 3.2 Outcome measures

#### 3.2.1 Primary outcome

The Emotional Functioning Outcome Scale (the Edinburgh Postpartum Depression Scale) will be used as primary outcomes to assess the severity of emotional disorders.

#### 3.2.2 Secondary outcomes

The secondary outcomes involved the Orientation to Life Questionnaire (sense of coherence 29-item scal) to assess tolerance of stress, the Hamilton Anxiety Inventory, and the Hamilton Depression Inventory to assess anxiety and depression, respectively. Notably, adverse events caused by acupuncture or other treatment therapies will be recorded.

#### 3.2.3 Search strategies

Firstly, eight databases will be searched for RCTs on acupuncture for PEDs, mainly including the PubMed, Web of Science, EMBASE, the Cochrane Central Register of Controlled Trials, Chinese National Knowledge Infrastructure, Chinese Biomedical Literature Database, Wanfang Database, and Technology Periodical Database. Terms of medical subject terms and keywords will be used individually or in combination in the query. The specific search strategy of PubMed is shown in Table 1. However, depending on the different database search methods, minor modifications to the terminology will be permitted. In addition, Chinese databases will be searched by using Chinese characters with synonym items.

**Table 1:** The search strategy used in PubMed database.

No.	Search items
#1	Randomized controlled trial [pt]
#2	Controlled clinical trial [pt]
#3	Randomized [tiab]
#4	Placebo [tiab]
#5	Clinical trials [MeSH]
#6	Randomly [tiab]
#7	Trial [ti]
#8	#1 OR #2 OR #3 OR #4 OR #5 OR #6 OR #7
#9	Humans [MeSH]
#10	#8 AND #9
#11	postpartum [MeSH]
#12	(Postnatal OR Perinatal OR after delivery OR after childbirth) [ti, ab]
#13	#11 OR #12
#14	Emotional disorder [MeSH]
#15	(Mood disorder OR affective disorder OR affective symptoms) [ti, ab]
#16	Depression [MeSH]
#17	(Depress OR Depressive OR dysthymia) [ti, ab]
#18	Anxiety [MeSH]
#19	Angst [ti, ab]
#20	Psychosis [ti, ab]
#21	post-traumatic stress disorder [ti, ab]
#22	obsessive-compulsive disorder [ti, ab]
#23	Nervousness [ti, ab]
#24	#14 OR #15 OR #16 OR #17 OR #18 OR #19 OR #20 OR #21 OR #22 OR #23
#25	Acupuncture therapy [MeSH]
#26	(Acupuncture OR electroacupuncture OR electroacupuncture therapy OR body acupuncture OR electro-acupuncture OR manual acupuncture OR auricular acupuncture OR warm needling) [ti, ab]
#27	#25 OR ##26
#28	#10 AND #13 AND #24 AND #27

MeSH = medical subject terms.

Secondly, clinical trial registries, such as the World Health Organization International Clinical Trials Registry, the Controlled Trials Meta-Registry, and the Chinese Clinical Trials Registry will be searched to analyze ongoing trials and unpublished data. Furthermore, relevant medical journals and magazines will be searched. Manual searches of potentially relevant references, including relevant systematic reviews, will be used to find further trials. And will attempt to contact the corresponding author to obtain clinical data that are not fully documented.

### 3.3 Data collection and analysis

#### 3.3.1 Collection of studies

**Figure 1:**
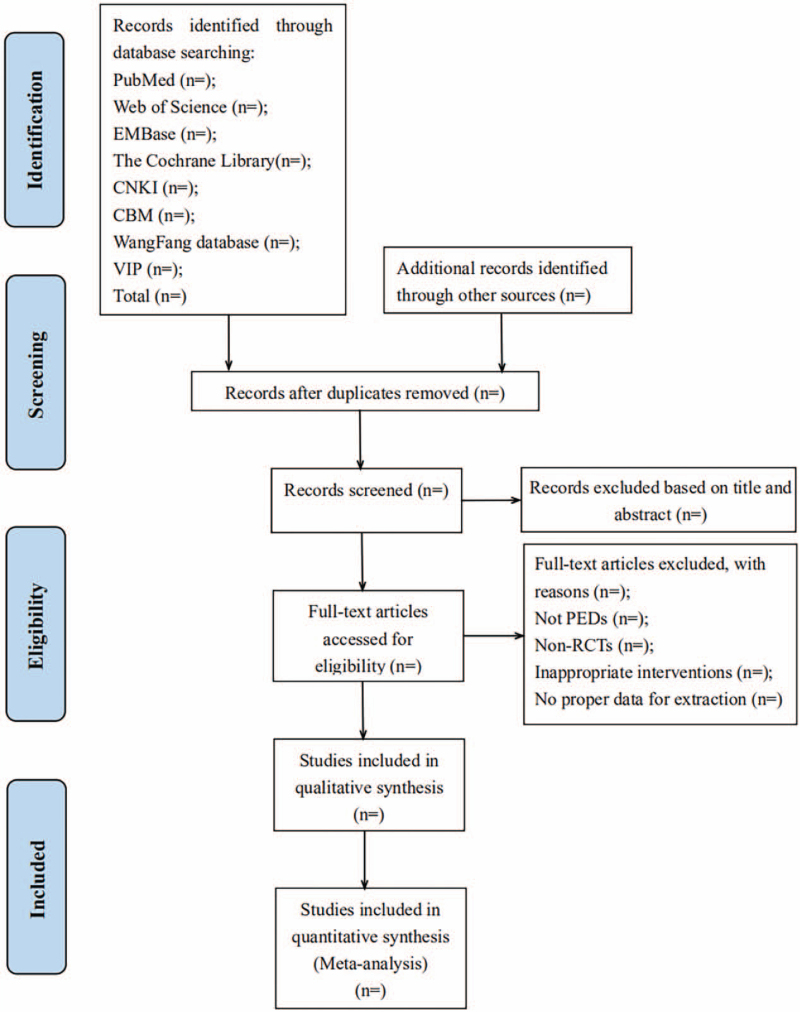
Flow diagram of the study selection process.

All potentially relevant literature will be searched independently by 2 researchers (NL and YYW). Any duplicate studies will be removed. Initial screening will be performed by title, abstract, and keywords, and after excluding irrelevant literature, the full text of all eligible studies will be downloaded for re-evaluation. During the evaluation process, any disagreement between two researchers about the eligibility of a document will be discussed with a third reviewer (JQF). Besides, the excluded articles and the reasons for their exclusion will be documented. The flow chart of literature screening is shown in Figure 1.

#### 3.3.2 Data extraction

Data will be extracted by 2 researchers (NL and YFX) independently and cross-checked by completing a designed data extraction form. The data extracted mainly includes

(1) Basic characteristics of the study (first author, time of publication, source/journal, institution, and country).
(2) Subject characteristics (diagnosis, baseline period status, sample size, mean duration of disease, mean age, inclusion criteria, and exclusion criteria).
(3) Intervention (type of acupuncture, randomization, allocation concealment, and blinding).
(4) Outcome (primary outcome, secondary outcome, adverse effects, and follow-up).
(5) Others (conflict of interest, ethical approval, etc).

Missing data will be obtained by contacting the corresponding author of the original article. Any disapproval will be decided by discussion or by a third reviewer (JQF).

#### 3.3.3 Dealing with missing data

All incomplete or missing data will be contacted by phone or e-mail to the corresponding author or the relevant authors. If there is no reply from the authors or missing data cannot be supplied, we will analyze it based on available information. The potential impact of missing data on the final results of the review will be shown in the discussion.

#### 3.3.4 Data synthesis

Data analysis will be performed by using RevMan 5.3.5 software. The method of measuring treatment effects will vary depending on the type of data. Dichotomous and continuous data will be analyzed using risk ratios with 95% confidence intervals and mean differences or standardized mean differences with 95% confidence intervals, respectively.

#### 3.3.5 Risk of bias in studies

Two authors (NL and YYW) will assess the risk of bias via the Cochrane Collaboration's tool for all included studies, including random sequence generation, allocation concealment, blinding, incomplete outcome data, and other sources of bias (eg, conflicts of interest, follow-up, etc). Each trial will be classified as low risk of bias, unclear (unclear or unknown risk of bias), or high risk of bias according to the criteria of the Cochrane guidelines.^[30]^ Any disagreement in the assessment process will be adjudicated by discussion or by a third reviewer (JQF).

#### 3.3.6 Quality of evidence assessment

The quality of evidence for each outcome will be assessed independently through the Grading of Recommendations Assessment, Development, and Evaluation system method,^[31]^ and the final report will be presented in four ratings: “high,” ‘medium’, ‘low’, or ‘very low’. Furthermore, the quality of evidence of a specific study will be assessed according to the basis of risk of bias, imprecision, inconsistency, indirectness, and reporting bias.

#### 3.3.7 Assessment of heterogeneity

Heterogeneity will be assessed by using the *I*^2^ statistical test. *I*^2^ < 50% indicates little or no statistical heterogeneity in these trials and a fixed-effects model will be used. And random-effects models will be used for *I*^2^ tests between 50% to 75%. Besides, if the *I*^2^ test is higher than 75%, qualitative or subgroup analyses will be performed and the corresponding explanations will be provided.

#### 3.3.8 Reporting bias

If there are more than 10 trials in the meta-analysis, primary outcome indicators will be examined qualitatively by funnel plots to analyze potential reporting bias as well as the impact of smaller studies.

#### 3.3.9 Sensitivity analysis

Sensitivity analyses will be used to test the robustness and reliability of the results and to assess the impact of methodological quality, sample size, and missing data. The results of the sensitivity analysis will be presented in summary tables. However, if all included studies have a high risk of bias, sensitivity analysis will not be performed.

#### 3.3.10 Subgroup analysis

Subgroup analyses will be conducted if sufficient data are available. Areas of subgroup analysis will primarily consider variation in subject and intervention characteristics, such as type of acupuncture and control interventions, differences in duration of onset of PEDs, and severity of emotional disorders.

#### 3.3.11 Ethics and dissemination

No ethical approval is required as the patient data will not be collected. In addition, the results of this meta-analysis will be disseminated through publication in peer-reviewed scholarly journals or relevant academic conferences to provide a reference for the effectiveness and safety of acupuncture treatment for PEDs.

## 4 Discussion

PEDs are a major public health problem that damages parent-child and family relationships, and has a non-negligible influence on the emotional, behavioral and cognitive development of children.^[32]^ Traditional pharmacological treatments or psychological interventions can reduce the severity of PEDs, whereas the side effects of drugs and the high cost of psychotherapy may reduce the efficacy and hinder the application.^[33,34]^ Acupuncture is becoming increasingly popular in western countries as a convenient, low-cost, safe, and superiorly effective treatment. In terms of reviews and meta-analyses were carried out to identify the efficacy of acupuncture in the treatment of PPD.^[35,36]^ Nevertheless, to the best our knowledge, PEDs related such as postpartum anxiety, posttraumatic stress disorder, obsessive-compulsive disorder, and severe psychosis could not be overlooked. Thus, this study is the first systematic review and meta-analysis of the effectiveness and safety of acupuncture in PEDs, looking forward to providing a high level of evidence-based for management of PEDs.

However, this study also has some limitations. The variance of acupuncture treatments and the different severity of PEDs will significantly lead to higher statistical heterogeneity. In addition, only Chinese and English publications will be included, which may lead to language bias.

To sum up, we sincerely hope that the results of this study will provide robust evidence for the efficacy and safety of acupuncture in the treatment of PEDs, to significantly inspire more peer experts to conduct large-sample randomized controlled studies in the future so that patients with PEDs will have a more optimal choice in terms of treatment methods.

## Acknowledgments

The authors would like to express their gratitude to all the advisors of this study.

## Author contributions

**Conceptualization:** Ning Luo, Yiyi Wang.

**Data curation:** Ning Luo, Yiyi Wang, Yunfan Xia.

**Formal analysis:** Ning Luo, Yiyi Wang, Yunfan Xia.

**Funding acquisition:** Yunfan Xia, Xiaomei Shao, Jianqiao Fang.

**Investigation:** Yunfan Xia.

**Methodology:** Ning Luo, Mingqi Tu, Xiaoting Wu.

**Software:** Mingqi Tu, Xiaoting Wu.

**Supervision:** Xiaomei Shao, Jianqiao Fang.

**Writing – original draft:** Ning Luo, Yiyi Wang.

**Writing – review & editing:** Ning Luo, Yunfan Xia.
